# The GenABEL Project for statistical genomics

**DOI:** 10.12688/f1000research.8733.1

**Published:** 2016-05-19

**Authors:** Lennart C. Karssen, Cornelia M. van Duijn, Yurii S. Aulchenko

**Affiliations:** 1 PolyOmica, Groningen, 9722 HC, Netherlands; 2 Department of Epidemiology, Erasmus Medical Center, Rotterdam, 3000 CA, Netherlands; 3 Institute of Cytology and Genetics, Siberian Division of the Russian Academy of Sciences, Novosibirsk, 630090, Russian Federation; 4 Novosibirsk State University, Novosibirsk, 630090, Russian Federation; 5 Centre for Global Health Research, Usher Institute of Population Health Sciences and Informatics, University of Edinburgh, Teviot Place, Edinburgh, EH8 9AG, UK

**Keywords:** Open source, Scientific software, Software development, Community building, Statistical genetics, Genomics, Statistical methodology

## Abstract

Development of free/libre open source software is usually done by a community of people with an interest in the tool. For scientific software, however, this is less often the case. Most scientific software is written by only a few authors, often a student working on a thesis. Once the paper describing the tool has been published, the tool is no longer developed further and is left to its own device. Here we describe the broad, multidisciplinary community we formed around a set of tools for statistical genomics. The GenABEL project for statistical omics actively promotes open interdisciplinary development of statistical methodology and its implementation in efficient and user-friendly software under an open source licence. The software tools developed withing the project collectively make up the GenABEL suite, which currently consists of eleven tools. The open framework of the project actively encourages involvement of the community in all stages, from formulation of methodological ideas to application of software to specific data sets. A web forum is used to channel user questions and discussions, further promoting the use of the GenABEL suite. Developer discussions take place on a dedicated mailing list, and development is further supported by robust development practices including use of public version control, code review and continuous integration. Use of this open science model attracts contributions from users and developers outside the “core team”, facilitating agile statistical omics methodology development and fast dissemination.

## Introduction

The field of statistical (gen-)omics lies at the heart of current research into the genetic aetiology of (human) disease and personalized or precision medicine ^1^. Genome-wide association studies (GWAS), genotype imputation and next-generation sequencing (NGS) are just a few of the techniques used in this field that is driven by increasingly larger data sets ^2, 3^. With the advent of polyphenotype analysis as is now customary in e.g. lipidomics and metabolomics, the issues of dealing with big data have become imminent ^4, 5^. In recent years, scientists and funding organizations alike have come to realize that in order to successfully tackle the challenges of the field, close collaboration between various disciplines, e.g. statistics, molecular biology, genetics, and computer science, is of paramount importance ^2, 6, 7^.

Many software tools developed by scientists are distributed as free/libre open source software (FLOSS). FLOSS tools are often developed by groups of people with different backgrounds, working from different geographical locations, either under central guidance or in a loose cooperation, sometimes as part of their employment, sometimes “just for fun” ^8^. The key to successful, sustainable open source software is an active community of both developers/contributors and end users ^9^. Unfortunately, creators of scientific software are usually not funded to actively build such a community. Moreover, our experience shows that once the peer-reviewed article describing a tool has been published, funding and time to continue development and support of that tool are usually limited or non-existent, and consequently, the tool often slowly fades into oblivion. It needs no explanation that this amounts to a waste of effort and money.

It was with these premises in mind that the *GenABEL Project for Statistical Genomics* was started as an extension of the original community of users and developers around the GenABEL package ^10^.

## The GenABEL project

**Figure 1.**
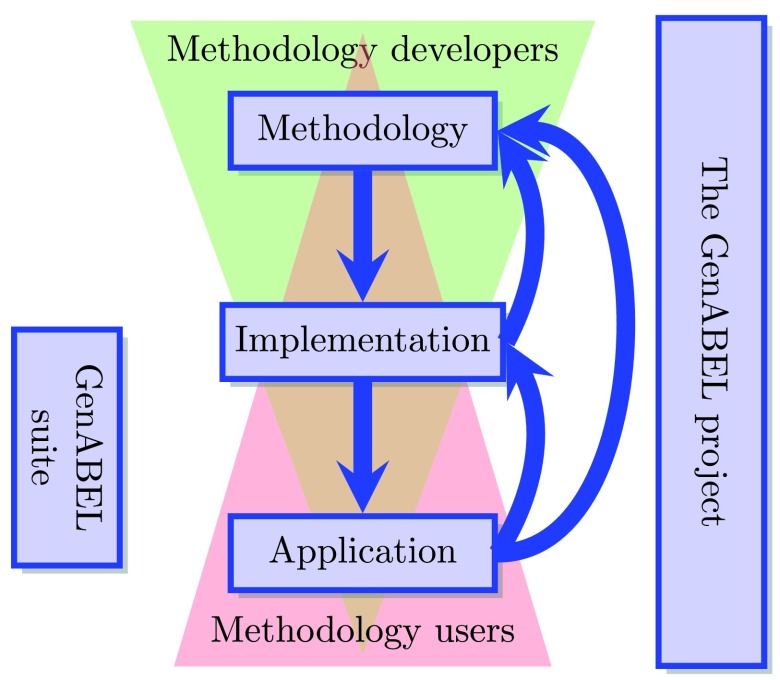
The structure of the GenABEL project and the information flow within it.

The GenABEL project aims to provide a framework for collaborative, sustainable, robust, transparent, opensource based development of statistical genomics methodology. Within the project, statisticians devoted to method development work together with statistical geneticists and biologists to refine existing statistical methods as well as develop new ones and make them applicable to genomic analysis. With the help of computer scientists and scientific software developers these mathematical models are then implemented into efficient and user-friendly software. This flow of work and information is not linear, but rather more circular in nature, with information and feedback being continuously transferred between the various layers as depicted in Figure 1. In short, it is a form of agile community-driven development ^11, 12^.

Openness is an important aspect of the GenABEL project ^13^. It enables a free flow of information between the layers in the project resulting in rapid feedback between the various levels. Not only do we require that all tools are released under an open source or free software licence like the GNU Public Licence (GPL), we also try to create an atmosphere of open communication using public mailing lists and web forums (see the sections Interaction with the user community and Development infrastructure below). Moreover, because of this openness results of the project (i.e. statistical methods as well as software packages) are easily disseminated among the end users, be they epidemiologists, bioinformaticians or others.

## The GenABEL suite

The software tools developed within the GenABEL project collectively make up the GenABEL suite. Many tools are R packages, however, this is not a requirement for inclusion in the suite. Any software that is related to the field of statistical (gen-)omics is welcome (technical requirements are discussed in section Development infrastructure). Currently, the suite consists of 11 officially released tools (cf. Table 1) and two that are in beta stage.

**Table 1.** The tools included in the GenABEL suite. Currently, all tools are licensed under the GNU Public Licence (GPL).

Tool	Year of first release	Year of latest release	Latest version
GenABEL ^10^	2007	2014	1.8-0
ProbABEL ^14^	2009	2016	0.5.0
MetABEL	2009	2014	0.2-0
DatABEL	2010	2015	0.9-5
MixABEL	2010	2015	0.1-3
ParallABEL ^15^	2010	2015	0.2-0
VariABEL ^16^	2011	2014	0.9-2
PredictABEL ^17^	2011	2014	1.2-2
OmicABEL ^18^	2013	2015	0.8.0
RepeatABEL ^19^	2015	2015	1.0
CollapsABEL ^20^	2016	2016	0.10.8

The GenABEL R package (not to be confused with the GenABEL project or the GenABEL suite), provides an efficient file format for storing genotype data and facilitates pre-GWAS quality control as well as running GWAS of continuous and binary phenotypes, and time-to-event data. The collaborative nature of the project is demonstrated in the GenABEL package as it implements several statistical methods developed within the framework, including approximate mixed models ^21– 23^ and various methods for genomic control ^24, 25^. This shows that the project is really a platform for implementation of (statistical) methods which removes the burden of thinking about data formats etc. allowing method developers to focus on what they do best. The GenABEL package is the most popular package in the GenABEL suite with more than 809 citations of its paper (according to Google Scholar) ^10^.

ProbABEL is a tool for running GWAS on imputed genotype data. Like the GenABEL package it allows running linear or logistic regression, as well as Cox proportional hazards model, however, ProbABEL is tailored to the large file sizes that are inherent to current data sets with approximately 30 million imputed genotypes per individual. It is the second most-used tool from the suite with more than 267 citations (according to Google Scholar) ^14^.

As indicated by its name, MixABEL is an R package for running genome-wide association analyses using mixed models in quantitative traits.

GWAS usually involves meta-analysis of the regression results of various cohorts. The R package MetABEL provides simple meta-analysis functions including generation of forest plots.

The R package VariABEL can be used to look for variance heterogeneity in genetic studies. Such heterogeneity is an indication of interaction between a genetic marker and either another marker or an unknown factor ^16, 26^.

In 2013 OmicABEL was added to the suite. It contains a high-performance computing based approach facilitating extremely fast mixed-model based regression of multiple omics traits like metabolomics or lipidomics on imputed genotype data ^18^. OmicABEL aims to increase computational throughput while reducing memory usage and energy consumption. This was achieved by using optimal (hardware-tailored) algorithms using state-of-the-art linear algebra kernels, incorporating optimizations and avoiding redundant computations.

PredictABEL is an R package for the assessment of genetic risk prediction models. It includes functions to compute univariate and multivariate odds ratios of the predictors, the area under the receiver operating characteristic (ROC) curve (AUC), Hosmer-Lemeshow goodness of fit test, reclassification table, net reclassification improvement and integrated discrimination improvement ^17^.

RepeatABEL allows one to run a GWAS for multiple observations on related individuals ^19^. Like ParallABEL, this package is a great example of contributions by the community since its development was not initiated by the core GenABEL developers.

CollapsABEL is the most recent addition to the GenABEL suite. It is an R library for detecting compound heterozygote (CH) alleles in GWAS. It is a computationally efficient solution for screening general forms of CH alleles in densely imputed microarray or whole genome sequencing datasets ^20^.

Apart from the aforementioned packages which directly address certain types of analysis and/or data management, several packages in the suite have a supportive role. DatABEL is an R interface to our filevector library which provides a file format that is optimised for fast access to data in matrix form, e.g. imputed genotype data. ParallABEL is an R library for parallel execution of GWAS in R.

The latest stable version of the R packages are available on CRAN ( http://cran.r-project.org), the Comprehensive R Archive Network. The source code for the other packages can be downloaded from our website at http://www.genabel.org, from the project’s version control server or on GitHub (see section Development infrastructure).

## Interaction with the user community

**Figure 2.**
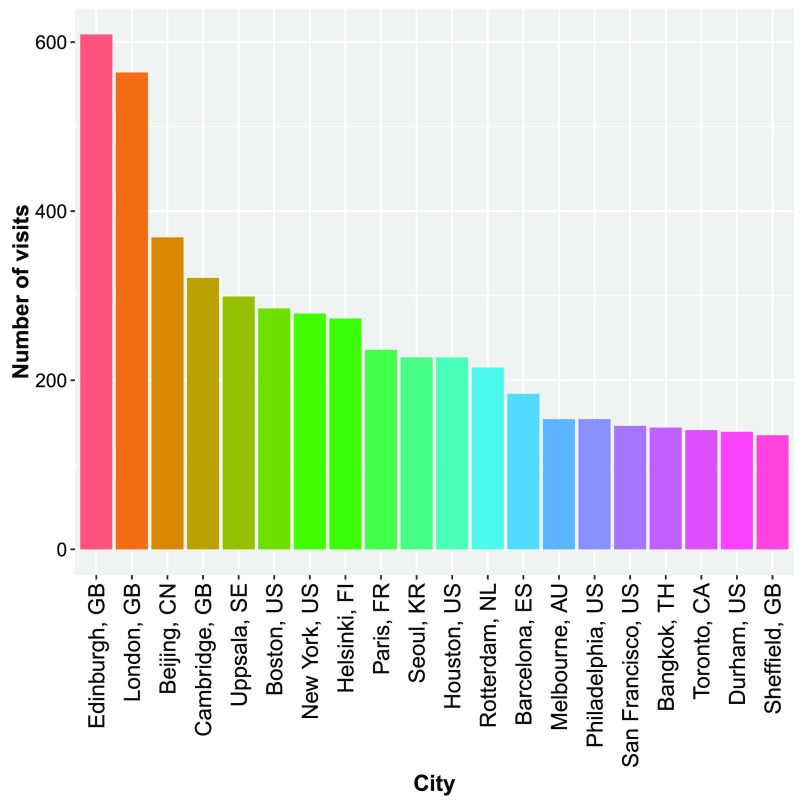
The top 20 cities of origin of visitors to the GenABEL website in the period 28 April 2015 – 28 April 2016. Only visits lasting more than 60 seconds and from cities from which more than 15 visits originated were taken into account. The total number of visits in that period was 16319, of which 696 came from unknown cities. Each city name is followed by the two-letter ISO code of the country in which it is located.

The GenABEL project website is the central hub that points to package descriptions, tutorials, the development website, and other information for potential and existing users and developers. Usage statistics such as number of visits and country of origin of visitors are monitored using Google Analytics ( http://www.google.com/analytics/) in order to get an estimate of the number of users of the tools and their origins. As an example of the information that can be obtained from this data, Figure 2 shows the top 20 cities of origin of the visitors of the GenABEL website in the period of 28 April 2015 till 28 April 2016. Only visits lasting more than 60 seconds and cities with more than 15 visits were taken into account in an attempt to filter out “accidental” visits. The website was visited 16319 times in that period, of which 696 visits were from an unknown city.

Collecting visitor data like this helps getting an insight in the institutes that use software from the GenABEL suite, which can then be used to show the impact the tools have, e.g. when applying for funding.

Interaction with the user community is done via social media like Twitter ( https://twitter.com/GenAproj) and Facebook ( https://www.facebook.com/pages/GenABEL-project/329281857167394), as well as a dedicated mailing list for announcements of new package releases, making it easy for both users and system administrators to keep up to date with new releases and developments in the GenABEL project and the GenABEL suite.

Each tool in the GenABEL suite has its own documentation and the GenABEL Tutorial ^27^ with more than 260 pages takes the user from learning basic R to performing more complicated analyses, showing how the various packages interconnect. Moreover, several video tutorials are available online.

Interactive user support is mostly done through our forum ( http://forum.genabel.org). Having an open forum serves various purposes. First of all it is a central, easy to point to reference. Moreover, compared to having individual users e-mailing a package author, who may be on holiday or otherwise unavailable, an open forum where users and developers collaborate helps in shortening the time-to-answer. Furthermore, having an active forum where users can help each other allows the developers to focus on fixing bugs and implementing new features. As of March 1st, 2015, the GenABEL forum has 538 activated user accounts, with an average 2.92 new registrations per week since the start of the forum in January 2011. These users have contributed 1422 posts in 427 topics, with an average 7.15 posts per week.

The first hurdle many users of (scientific) software encounter is the installation process. Within the GenABEL project we aim to make installation as simple as possible. Using CRAN for the R packages makes installation and upgrading as simple as typing a single command. For the tools that don’t use R, we aim to provide up-to-date packages for various Linux distributions. Currently, ProbABEL is packaged in the Stable, Testing and Unstable repositories of Debian with the help of the Debian Med team ^28^. Other packages are planned to be added before the end of 2016. For Ubuntu Linux a Personal Package Archive is available ( https://launchpad.net/~l.c.karssen/+archive/genabel-ppa). Packages for Red Hat Enterprise Linux and CentOS are on the road map, but haven’t been released yet.

## Development infrastructure

The GenABEL project welcomes contributions of all sorts, from new tools to fixing spelling errors in the documentation, to bug reports and feature requests. To this end all program code and documentation are either stored in a publicly readable instance of the Subversion version control system, with write access limited to a group of core contributors, or on GitHub ( https://github.com/GenABEL-Project), which is one of the leading platforms for what is termed “social coding”, which perfectly fits the project’s goals. These version control systems record any change to the files so they can easily be reviewed and reverted if necessary ^7, 29, 30^.

In November 2010 a mailing list was created as a central place for development discussions. As of April 2016 this list has 34 subscribers. The GenABEL development website ( https://r-forge.r-project.org/projects/genabel/) including the Subversion server, the mailing lists, and the trackers for bugs and feature requests are kindly provided by the R-Forge project ^31^. Currently, a total of 94 bugs have been submitted to the bug trackers on R-forge and GitHub since their opening in 2010 and 2015, respectively. Of these 94, 12 were directly contributed by people outside of the core team of developers. Another 42 bug reports were filed by regular contributors based on user reports on the forum, for example, which means that 56% of the bugs have been reported by people in our community that are not core contributors.

In order to be able to maintain the quality of both old and new software in the GenABEL suite prospective tools go through a review process in which both the functional quality of the code is evaluated (does the tool do what it intends to do?), as well as the actual quality of the code (is the code clearly written, including developer documentation in the form of e.g. comments; does the code conform to the GenABEL coding style guidelines; etc.). Moreover, as set out in the GenABEL developer guidelines, we expect commitment of the person or team submitting a tool to the suite to maintain and support it, otherwise the maintenance burden would end up with the core team and it would be too easy to create a tool, write a paper and then ‘dump’ it in the GenABEL project hoping “the community” will take care of it. Therefore, the community has the option to mark a tool as obsolete, warning the user that bugs will no longer be fixed and support is limited or non-existent.

In 2013 we have started to use a Jenkins Continuous Integration server. Using Jenkins various tests (e.g. regression tests, build tests and tests for memory leaks) are automatically run on each commit to the version control systems. Consequently, changes that break existing functionality are detected at an early stage, thus leading to more stable software releases.

## Conclusion

The original publication of the GenABEL package for statistical analysis of genotype data ^10^ has led to the evolution of a community which we now call the GenABEL project, which brings together scientists, software developers and end users with the central goal of making statistical genomics work by openly developing and subsequently implementing statistical models into user-friendly software.

The project has benefited from an open development model, facilitating communication and code sharing between the parties involved. The use of a free software licence for the tools in the GenABEL suite promotes quick uptake and widespread dissemination of new methodologies and tools. Moreover, public access to the source code is an important ingredient for active participation by people from outside the core development team and is paramount for reproducible research. Feedback from end users is actively encouraged through a web forum, which steadily grows into a knowledge base with a multitude of answered questions. Furthermore, our open development process has resulted in transparent development of methods and software, including public code review, a large fraction of bugs being submitted by members of the community, and quick incorporation of bug fixes.

## Data and software availability

The file tracker_report-2016-04-16.csv contains the data exported from the GenABEL R-forge bug tracker as it was on the date listed in the file name. Because of the recent move of some of the tools from R-forge to Github, the number of issues on the Github pages of the GenABEL project was still low. Therefore, these were counted manually.

The file Analytics www.genabel.org Locatie Lennart 20150428-20160428.csv contains the data extracted from the Google Analytics page for the GenABEL website for the period listed in the file name. The columns contain the ISO code of the country, city, number of sessions, number of new viewers, bounce percentage, pages per session and average session duration, respectively.

The file analysis_GenABELpaper.org contains the source code used for the automated data extraction for this paper in Emacs Org mode literate programming format ( http://orgmode.org) ^32^.

The code contained in the Org mode file and the data in the csv files listed above are in the public domain (Creative Commons CC0 license) and can be used without restriction.

The data related to the GenABEL forum were extracted manually from the forum control panel.

The tools currently in the GenABEL suite are all Free Software, licensed under the GNU Public License. An up-to-date list of the packages in the suite can be found on http://www.genabel.org/packages, which also contains pointers to the source code of the latest stable versions and the version control repositories on R-forge and GitHub (see the section Development infrastructure above for the URLs).

**Archived source code at the time of publication** https://zenodo.org/record/51008 ^33^
