# Supplementary material for: The GenABEL Project for statistical genomics
Source: F1000Res. 2016 May 19;5:914. [Version 1] doi: 10.12688/f1000research.8733.1 (PMC4916982; doi:10.12688/f1000research.8733.1)
Supplement: Peer review history

# Peer review: Referee response for version 1

**DOI:** 10.5256/f1000research.9397.r14125

**Published:** 2016-06-17

**Authors:** Bjarni J. Vilhjálmsson

**Affiliations:** 1 Bioinformatics Research Centre, Aarhus University, Aarhus, Denmark

Karsen *et al. *presents a paper on the GenABEL project, which is really a software suite that contains 11 different packages. This is a large and impressive project that contains packages that are routinely used by researchers studying genetics. Indeed, this is reflected by more than 800 citations (according to google scholar) for the original GenABEL paper published in 2006. However, the 11 packages presented in this paper have been published previously in some shape or form, albeit (presumably) not in their most recent version. It is therefore tempting to question the novelty of the current paper that summarizes the GenABEL project and describes its user and developer community. Nevertheless, despite limited amount of novel scientific ideas or scientific results in the current manuscript, the authors have clearly put a lot of work into creating a very impressive interactive user and developer community. Furthermore, the paper is well written and it will undoubtedly be highly cited by future researchers. Lastly, to reiterate, the GenABEL is a very impressive large scale project that is heavily used by the community! I therefore think this is overall a nice publication that is certainly suitable for indexation.

 Minor comments:

- Abstract: “…developed withing..” -> “…developed within..”
- I think it would be magnanimous if you acknowledged contributors as authors, e.g. under an umbrella term “The GenABEL community”.
- Leave citations counts out of manuscript, it looks awkward.  They will also change.
- On p. 3 “polyphenotype”, I think multivariate would be a better word.

I have read this submission. I believe that I have an appropriate level of expertise to confirm that it is of an acceptable scientific standard.

# Peer review: Referee response for version 1

**DOI:** 10.5256/f1000research.9397.r14057

**Published:** 2016-05-27

**Authors:** Giulietta Minozzi

**Affiliations:** 1 Department of Veterinary Science and Public Health, University of Milan, Milan, Italy

This very well written article describes the GenABEL project for statistical genomics and high lightens the great success of the project, that in the years has lead to the creation of an actual scientific community that is spread in several countries worldwide.

 It is my understanding that the manuscript is aimed at reaching potential new users, but even to old users, unaware of the possible new tools included in the GenABEL project.

 The methodologies underlining the different tools and their potential applications are well described. Figure 2 describes the extent of the use of the GenABEL website/suite and Table 1 gives a glance of the tools available and on the new versions implemented.

 It is clear, from the texts, the effort that has taken place and is taking place aiming at bringing together top scientists, software developers and end users with the central goal of making statistical genomics work by openly developing and subsequently implementing statistical models into a user-friendly software.

 I retain this article, the tools and the information provided of extreme importance to the scientific community.

I have read this submission. I believe that I have an appropriate level of expertise to confirm that it is of an acceptable scientific standard.
